# Making Every “Point” Count: Identifying the Key Determinants of Team Success in Elite Men’s Wheelchair Basketball

**DOI:** 10.3389/fpsyg.2019.01431

**Published:** 2019-07-02

**Authors:** John Francis, Alun Owen, Derek. M. Peters

**Affiliations:** ^1^School of Sport and Exercise Science, University of Worcester, Worcester, United Kingdom; ^2^Faculty of Engineering, Environment and Computing, Coventry University, Coventry, United Kingdom; ^3^School of Allied Health and Community, University of Worcester, Worcester, United Kingdom; ^4^Faculty of Health and Sport Sciences, University of Agder, Kristiansand, Norway

**Keywords:** sport performance analysis, Paralympic, European championships, logistic regression, predictive modeling

## Abstract

Wheelchair basketball coaches and researchers have typically relied on box score data and the Comprehensive Basketball Grading System to inform practice, however, these data do not acknowledge how the dynamic perspectives of teams change, vary and adapt during possessions in relation to the outcome of a game. Therefore, this study aimed to identify the key dynamic variables associated with team success in elite men’s wheelchair basketball and explore the impact of each key dynamic variable upon the outcome of performance through the use of binary logistic regression modeling. The valid and reliable template developed Francis et al. (2019) was used to analyze video footage in SportsCode from 31 games at the men’s 2015 European Wheelchair Basketball Championships. The 31 games resulted in 6,126 rows of data which were exported and converted into a CSV file, analyzed using R (R Core Team, 2015) and subjected to a data modeling process. Chi-square analyses identified significant (*p* < 0.05) relationships between Game Outcome and 19 Categorical Predictor Variables. Automated stepwise binary regression model building was completed using 70% of the data (4,282 possessions) and produced a model that included 12 Categorical Predictor Variables. The accuracy of the developed model was deemed to be acceptable at accurately predicting the remaining 30% of the data (1,844 possessions) and produced an area under the receiver operating characteristic curve value of 0.759. The model identified the odds of winning are more than double when the team in possession are in a state of winning at the start of the possession are increased five-fold when the offensive team do not use a 1.0 or 1.5 classified player, but are increased six-fold when the offensive team use three or more 3.0 or 3.5 players. The final model can be used by coaches, players and support staff to devise training and game strategies that involve selecting the most appropriate offensive and defensive approaches when performing ball possessions to enhance the likelihood of winning in elite men’s wheelchair basketball.

## Introduction

Wheelchair basketball is a very popular disability sport (Sporner et al., 2009), with over 105 nations registered with the sport’s international body, the International Wheelchair Basketball Federation [IWBF] (2019b). The rules of wheelchair basketball are very similar to running basketball albeit with basic rule adaptions to meet the needs of playing the game in a wheelchair, and with the primary objective of scoring more points than their opponents (International Wheelchair Basketball Federation [IWBF], 2019a). In an attempt to ensure fair and equitable competition the IWBF introduced a “Functional Player Classification System” in 1984 to assess a player’s functional capacity to push, pivot, shoot, rebound, dribble, pass and catch (International Wheelchair Basketball Federation [IWBF], 2014a). The current classification system comprises of eight sport classes (Classes 1.0, 1.5, 2.0, 2.5, 3.0, 3.5, 4.0 and 4.5) with half-point classes being used for borderline cases. As the level of an individual’s functional ability increases so does the individual’s level of classification, with those players with a Class 4.5 being described as those individuals with the least eligible impairment. During a game, the maximum points of the five on-court players per team must not exceed 14 points.

With the growth in the sport, the performance gap between participation and qualification into a World Championships or Paralympic Games has increased and resulted in teams becoming more strategic in the way athletes and teams prepare for competitions (de Bosscher et al., 2008). One of the newest sports science disciplines to be used in wheelchair basketball, in collaboration with coaches’ knowledge, involves the labeling and recording of sports specific actions and behaviors and is referred to as performance analysis (Sampaio et al., 2013). Despite performance analysis research in wheelchair basketball being published since 1995, wheelchair basketball programs have only recently employed performance analysts to bridge the gap in the coaches’ knowledge and unlock objective marginal gains. This finding continues to highlight the frequent disconnect between research and the application of the findings into practice due to a lack of situation-specific context (Hughes and Franks, 2004; Mackenzie and Cushion, 2013).

Vanlandewijck et al. (1995) were the first researchers to use box-score data from performances during the 1992 Paralympic Games to explore the relationship between classification and on-court performance. Boxscore data was used to present a summary of an individual’s in-game performance and made use of 13 specific actions and behaviors. The box score data was used to evaluate an individual’s quality in relation to game performance through the Comprehensive Basketball Grading System (CBGS) (Byrnes, 1989). The system considered the following variables when calculating an individual’s performance, and assigned a total score and an average score based on the minutes played: “field goals made (+5), field goals attempted (–2), free throws made (+5), free throws attempted (–2), offensive rebound (+3), defensive rebound (+2), loss of ball possession (–5), personal and technical fouls (–5), assists (+5), turnovers (–5), blocked shots (+3), steals (+5) and forced turnovers on defense (–5)” (Vanlandewijck et al., 1995, p. 141).

Vanlandewijck et al. (1995) reported that an individual’s game efficiency is dependent on their classification, however, no variables were included to consider the individual’s disability nor the skills of players in a wheelchair. Attempts were made by Vanlandewijck et al. (2003, 2004) to address this issue through the development of a modified CBGS, but the following variables were removed due to a misunderstanding in the operational definitions: back picks, forced turnovers in defense, and both fouls. Subsequently, further performance analysis research in wheelchair basketball (Molik and Kosmol, 2001; Molik et al., 2009) has also elected to only use 12 instead of the 16 action variables proposed by Byrnes and Hedrick (1994) (Offensive Rebound, Defensive Rebound, Steals, Blocked Shots, Assists, Free-Throws Made, Two-Point Shots Made, Three-Point Shots Made, Free-Throws Missed, Two-Point Shots Missed, Three-Point Shots Missed and Turnovers). These variables, referred to as discrete action variables, lack contextually specific information and do not provide researchers or coaches with an understanding of the dynamic actions completed by individual player’s and team’s that occur in game or training performances.

More recent research in performance analysis completed by Gómez et al. (2014). Gómez et al. (2015a) has combined the individual player box-score data of teams, with situational variables, in an attempt to provide an objective insight into team performance in relation to the outcome of a game and address the lack of situation-specific context. According to Gómez et al. (2014), the data and calculations have the potential to evaluate a team’s performance and inform the decisions coaches are required to make during games. However, the studies still used discrete action variables which do not provide insight into how or why an individual’s or team’s action occurred. If coaches are relying on data collected from individual player discrete action variables to inform their team decisions, the information is one-dimensional and does not acknowledge how the dynamic perspectives of teams change, vary and adapt during possessions in relation to the outcome of a game. Subsequently, this collected discrete action variable data were argued to be both inaccurate and unreliable to inform future team strategic actions (Ziv et al., 2010).

Within wheelchair basketball, the actions and subsequent changes in the offensive and defensive strategies can be recorded to identify reoccurring patterns. Garganta (2009) found that players only have a set number of available options to them in any given situation, which is influenced by the interactions and actions of the opposing team. Therefore, it is possible to record these patterns and identify reoccurring trends to enhance the understanding of a team’s tactics (Perl et al., 2013). The sequential data, that includes situational action variables, such as the state of the game or which players are on the court, enables a greater understanding of the actions within a possession to be gained in relation to the end result of a game (Game Outcome: win or lose). For example, if the offensive team have been shown to advance the ball toward the basket quickly following a turnover when there are two 2.0 or 2.5 players on the court, then, the defensive team’s coaches can address this and inform their players to unsettle the offensive team by adjusting the defensive system when a turnover occurs. The change in how the possession starts could be measured in relation to the offensive team’s adjustments and the subsequent actions of the defensive team. It is these decisions and adjustments by the offensive and defensive team that can be collated, analyzed and interpreted in relation to the outcome of the game. Thus, the findings can be used to inform future decisions by exploring how and why a specific incident occurred (Kubatko et al., 2007).

Through the utilization of modeling techniques, the effect of each sequential action variable on the odds of Game Outcome can be calculated. For example, Gómez et al. (2013) used binary logistic regression modeling to identify the key action variables associated with achieving success in basketball and quantified the effect of each action variable on the “Game Outcome.” The insights gained from this type of modeling can be used to assist coaches, players and members of support staff with understanding the potential positive or negative impact on game outcome based on the decisions they have made. The data can be used to assist decisions around the planning and delivery of training sessions as well as informing in-game decisions through exploiting performance factors which are most highly associated with success (Petersen et al., 2007; Passos, 2017). The use of binary logistic regression modeling in wheelchair basketball may, therefore, enhance the understanding of the coaches, players and support staff regarding the key requirements of the game and provide objective insight into the effect of individual action variable on the odds of the Game Outcome.

By collecting, analyzing and modeling performance data in this way it may be possible to identify reoccurring themes and trends within a team’s performance. Francis et al. (2019) overcame the limitations of existing performance analysis templates in wheelchair basketball and developed a template to collect valid and reliable team performance data. The observers within the study were able to accurately notate the observed sequential actions of an elite game of wheelchair basketball regardless of performance analysis experience or wheelchair basketball knowledge. The researchers argued that the template has the potential to collect data concerning the interaction between offensive tactics (e.g., taking a shot within 6 s of being in possession) and defensive tactics (e.g., Press vs. Highline vs. Zone defense), acknowledging the effects of various different classification line-ups throughout the course of a single game or across multiple games. If significant trends are discovered, the data have the potential to identify the key components of success. The data would provide insights into the impact each action variable has on success and enable analysts and performance staff to contextualize a performance, answering the question of how and why a behavior or specific action occurred (Clemente et al., 2016). This information can be used to inform the decision-making of coaches, players and support staff (Busemeyer and Pleskac, 2009). Therefore, the aims of this paper were to use Francis et al. (2019) template to (i) identify the key dynamic variables associated with team success in elite men’s wheelchair basketball and (ii) explore the impact of each key dynamic variable upon the outcome of a performance through the use of binary logistic regression modeling.

## Materials and Methods

### Sample

The sample consisted of 6,126 possessions from the performances of 12 national teams that participated in the 2015 European Wheelchair Basketball Championships (Great Britain – 1st; Turkey – 2nd; Germany – 3rd; Netherlands – 4th; Spain – 5th; Italy – 6th; Poland – 7th; Israel – 8th; Sweden – 9th; Switzerland – 10th; France – 11th; Czech Republic – 12th). The tournament was a qualification event for the 2016 Rio de Janeiro Paralympic Games and the European Zone were given five qualification spots for the 2016 Paralympic Games and these were given to Germany, Great Britain, Netherlands, Spain and Turkey. A total of 31 games out of a total of 46 games were selected for the study (Table 1). The criteria for inclusion was that a game had to involve one of the qualified nations. Following the granting of ethical approval from the University of Worcester’s Ethics and Research Governance Committee, written, voluntary informed consent, was obtained from the host nation to access the required game footage.

**TABLE 1:** Summary of the number of games included in the sample per team.

**Team**	**Number of games**	**Number of possessions**
Great Britain	8	788
Turkey	8	713
Germany	8	749
Netherlands	8	739
Spain	8	872
Italy	4	404
Poland	5	472
Israel	3	316
Sweden	2	201
Switzerland	2	217
France	3	321
Czech Republic	3	334

**Total**	**62 (31 unique games)**	**6,126**

### Variables, Performance Analysis Template and Reliability

The new reliable performance analysis template for quantifying action variables in elite men’s wheelchair basketball developed by Francis et al. (2019) was utilized for analyzing the sample of games. The template included 109 action variables that were placed into 17 agreed Categorical Predictor Variable (CPV) categories by four wheelchair basketball staff and the lead researcher (Table 2). Francis et al. (2019) reported weighted kappa coefficients and percentage error values, for both intra-observer (<K0.980; <1.50%) and inter-observer (<K0.974; <3.00%) testing, that fell within the agreeable thresholds (Cohen, 1968; Bland and Altman, 1999).

**TABLE 2:** Summary of the 109 action variables within the 17 categorical predictor variables.

**Categorical predictor variables**	**Action variable**	**Categorical predictor variables**	**Action variable**
Quarter	Quarter 1	Shot Taken	Shot
	Quarter 2		No Shot
	Quarter 3	Shot Outcome	Successful
	Quarter 4		Unsuccessful
	Over Time		No Shot
Game Status	Winning	Shot Point	One
	Drawing		Two
	Losing		Three
Home Team	4		No Shot
	5	Shot Clock Remaining	6–0.1 s
	6		12–7 s
	7		17–13 s
	8		24–18 s
	9		Dead
	10		No Shot
	11	Shot Location	2 Point – Left – Base
	12		2 Point – Left – 45
	13		2 Point – Left Elbow
	14		2 Point – Centre – Near
	15		2 Point – Centre – Mid
Away Team	4		2 Point – Centre – Long
	5		2 Point – Right – Base
	6		2 Point – Right – 45
	7		2 Point – Right Elbow
	8		3 Point
	9		Free Throw Line
	10		No Shot
	11	Man Out Offence	Equal Numbers
	12		Numbers Advantage
	13	Defensive Outcome	Successful Defense
	14		Unsuccessful Defense
	15	Possession	Maintained
Home Classification	1		Lost
	1.5		Basket Scored
	2	End of Possession	Foul Against
	2.5		Foul For
	3		Violation Against
	3.5		Defensive Rebound
	4		Offensive Rebound
	4.5		Basket Scored
Away Classification	1		Other
	1.5		Out of Bounds
	2		Free Throw
	2.5		Handling Error
	3	Defensive System	1 Man Press
	3.5		2 Man Press
	4		3 Man Press
	4.5		4 Man Press
Start of Possession	Inbound – Baseline		5 Man Press
	Inbound – Endline		Highline
	Sideline – Front		Zone
	Sideline – Back		No Defensive System
	Defensive Rebound		
	Offensive Rebound		
	Free Throw		
	Other Start		
	Turnover		

### Data Collection and Handling Procedure

The obtained game footage was filmed from a half-way elevated position and provided a half-court perspective with an overlay of the time clock and current scoreboard. The 31 games were analyzed over a two-month period at the end of 2015 by the lead author using the template developed by Francis et al. (2019). The lead author had been part of the template development process and spent an average of 2 h to analyze each game. On any given day, a maximum of two games were analyzed in an attempt to reduce errors and a five-minute break was taken at the end of each quarter (Liu et al., 2015). Periodic assessment checks were conducted in an attempt to limit the overall loss of accuracy (Kazdin, 1977). Following the analysis of every five games, 10 randomly selected possessions were re-observed to identify any discrepancies. No adjustments to the analyzed data were necessary.

Following data collection in SportsCode, the data were exported into Microsoft Excel using the “Sorter” function in SportsCode. The 31 games resulted in 6,126 rows of data, each of which related to a single ball possession consisting of 17 columns (one for each CPV referred to earlier). Additional information relating to Possession Number, Game Outcome and Stage of Competition were also added to the data, making the dataset consisting of 20 columns. Each row was subjected to data cleaning to identify any discrepancies within the data. If any missing or duplicated data were identified, the game and possession were identified and re-analyzed. In total, four possessions were re-analyzed to input missing data.

The Home Classification and Away Classification columns from the dataset were reformatted into eight new columns to demonstrate the number of each classification from the offensive and defensive team were involved in a possession. For example, if the offensive team comprised of two 1.0 players and three 4.0 players, the column Offensive Unit – 1.0–1.5 would present as Two, the Offensive Unit – 4.0–4.5 column would show Three, whilst both the Offensive Unit – 2.0–2.5 and Offensive Unit 3.0–3.5 would present as Zero. Likewise, if the defensive unit comprised of one 1.0 player, three 3.0 players and a 4.0 player, the column Defensive Unit – 1.0–1.5 would present as One, the Defensive Unit – 3.0–3.5 column would show Three, the column Defensive Unit – 4.0–4.5 would present as One, whilst the Defensive Unit – 2.0–2.5 would present as Zero. Through making this adjustment, the data could be used to explore whether there is an optimum unit combination that could be used dependent upon the opposition’s line-up unit.

Following checking and sorting of the data and removal of redundant columns in the data set, it now consisted of 23 columns: Defensive Outcome, Defensive System, Defensive Unit – 1.0–1.5, Defensive Unit – 2.0–2.5, Defensive Unit – 3.0–3.5, Defensive Unit – 4.0–4.5, End of Possession, Game Status, Man Out Offence, Offensive Unit – 1.0–1.5, Offensive Unit – 2.0–2.5, Offensive Unit – 3.0–3.5, Offensive Unit – 4.0–4.5, Possession Outcome, Quarter, Shot Clock Remaining, Shot Location, Shot Outcome, Shot Point, Shot Taken, Stage, Start of Possession and Game Outcome (Dependent Variable). Recall that the title of each of the columns (apart from Game Outcome) is referred to as a CPV (Categorical Predictor Variable) and that the action variables within each CPV are referred to as action variables. The Excel file was converted into a CSV file (Supplementary Material S1) and subjected to statistical analysis procedures.

### Statistical Methods

First, cross-tabulations were created to ensure all action variables within a CPV had a greater frequency of 40, such that any action variable that reported a low-frequency count was merged with other suitable action variables. Second, Pearson chi-square analysis was carried out to determine if there was an association between each independent variable (i.e., each CPV) and the dependent variable, “Game Outcome.” Cramer’s V was subsequently used as a post-test to determine the strength of any observed association. Third, assessment of inter-associations and multicollinearity were conducted, with any CPV reporting a variance inflation factor above the threshold of 10 (Myers, 1990) being removed. Therefore, the following CPVs were excluded from multivariable analyses: Defensive Outcome, Man Out Offence, Possession Outcome, Quarter, Shot Taken and Shot Location. Fourth, a binary logistic regression model was developed through an automated stepwise approach (Hosmer and Lemeshow, 2000) using 70% of the data (training sample) (Dobbin and Simon, 2011), which attributed to 4,282 possessions. The dependent variable used in the model was Y = 0, 1, … with 0 indicating a loss and 1 indicating a win for the game outcomes. Then, the binomial logistic regression model can be expressed as the expected value if Y given the data (*X*) for any individual possession as follows:

$$E\,\left(Y|X\right)=\frac{e^{\left(Z\right)}}{1+e^{\left(Z\right)}}$$

where *Z* represents

$$\begin{array}{c} \beta 0+\beta 1\times \mathrm{GS}+\beta 2\times \mathrm{ST}+\beta 3\times \mathrm{O1}+\beta 4\times \\ \mathrm{O3}+\beta 6\times \mathrm{O4}+\beta 7\times \mathrm{D1}+\beta 8\times \\ \mathrm{D2}+\beta 9\times \mathrm{D3}+\beta 10\times \mathrm{D4}+\beta 11\times \\ P+\beta \,12\times \mathrm{EP}+\beta \,13\times \mathrm{DS} \end{array}$$

where β0 is the constant of the equation and the independent variables were the CPVs: GS = Game Status, ST = Stage, O1 = Offensive Unit – 1.0–1.5, O2 = Offensive Unit – 2.0–2.5, O3 = Offensive Unit – 3.0–3.5, O4 = Offensive Unit – 4.0–4.5, D1 = Defensive Unit – 1.0–1.5, D2 = Defensive Unit – 2.0–2.5, D3 = Defensive Unit – 3.0–3.5, D4 = Defensive Unit – 4.0–4.5, SP = Start of Possession, EP = End of Possession, DS = Defensive System. To explore the individual contributions of each action variable within the binary logistic regression model, the estimated regression coefficients and their standard error values along with their *p*-values, Odds Ratio (OR) values and their 95% confidence intervals (CIs) were determined. The estimated regression coefficients demonstrated the action variables’ contribution to the prediction of the outcome (game success), with a positive estimated regression coefficient being associated with an increase in the odds of winning the game.

The fifth and final stage involved exploring the fit of the model. McFadden (1974), Cox and Snell (1989), and Nagelkerke (1991) pseudo-*R*^2^ values were determined to indicate the degree in which the model explained the amount of the variation in game outcomes. To calculate the model’s ability to accurately predict out of sample game outcomes, the area under the receiver operating characteristic (ROC) curve (Hosmer and Lemeshow, 2000) was used to compute the sensitivity and specificity of the model against the remaining 30% of data (testing sample (1,844 possessions). The five stages of statistical analyses were performed using the software R (R Core Team, 2015), version 3.4.2, with the level of significance set at *p* < 0.05. The following packages were used: “car” package [Companion to Applied Regression (car): Fox et al. (2018)], the “caret” package [Classification and Regression Training (caret): Kuhn et al. (2018)], the “scales” package [Scale Functions for Visualization (Wickham, 2018)] and the “ROCR” package (Sing et al., 2015).

## Results

A Pearson chi-square test of independence was performed to examine the association between Game Outcome and 22 CPVs. The relationship between 19 of these CPV was found to be significant (*p* < 0.05) (Table 3). Cramer’s V measure of nominal association showed that when a team was in a status of winning in comparison to losing or drawing the team were more likely to win the game (ϕ_*C*_ = 0.362, *p* < 0.001). In addition, teams were significantly more likely to win a game when the offensive team comprised of two players who were classified as either a 2.0 or a 2.5 player (ϕ_*C*_ = 0.116, *p* < 0.001). Furthermore, when the offensive team comprised of two 3.0 or 3.5 players the likelihood of winning the game increased (ϕ_*C*_ = 0.131, *p* < 0.001). Whilst, if the defensive team comprised of either zero or two 3.0 or 3.5 players the likelihood of the offensive team winning increased (ϕ_*C*_ = 0.173, *p* < 0.001).

**TABLE 3:** Chi-square tests of association with game outcome and each individual CPV.

**CPV**	***χ*^2^**	***df***	***p***
Defensive Outcome	39.58	1	<0.001
Defensive System	37.94	7	<0.001
Defensive Unit – 1.0**–**1.5	32.46	2	<0.001
Defensive Unit – 2.0–2.5	56.08	2	<0.001
Defensive Unit – 3.0–3.5	182.36	3	<0.001
Defensive Unit – 4.0–4.5	20.03	2	<0.001
End of Possession	53.84	10	<0.001
Game Status	803.49	2	<0.001
Man Out Offence	0.06	1	0.801${}^{∼}$
Offensive Unit – 1.0–1.5	19.05	2	<0.001
Offensive Unit – 2.0–2.5	82.63	2	<0.001
Offensive Unit – 3.0–3.5	104.77	3	<0.001
Offensive Unit – 4.0–4.5	69.12	2	<0.001
Possession	50.78	3	<0.001
Quarter	0.81	3	0.848^∼^
Shot Clock Remaining	14.14	4	<0.001
Shot Location	43.91	10	<0.001
Shot Outcome	10.40	2	0.005
Shot Point	26.14	3	<0.001
Shot Taken	0.51	1	0.473^∼^
Stage	76.09	4	<0.001
Start of Possession	65.91	8	<0.001

*^∼^Denotes CPV was removed from further analyses due to a non-significant relationship being found.*

In a binary logistic regression model, the Odds Ratio (OR) represents a measure of association between an independent variable and an outcome variable (Szumilas, 2010). If an OR of greater than one is found for an action variable, this describes a positive relationship and means that if this action variable occurs it is associated with higher odds of winning. Whereas, if an OR of less than one is found for an action variable, this describes a negative relationship, and means that if this action variable occurs it is associated with lower odds of winning. Therefore, if an action variable was included in the model and has an OR greater than one, the odds of winning the game increased. The results of the binary logistic regression (Table 4) showed the influence of Game Status, the offensive and defensive line-up combinations, how possession started and ended as well as the defensive system operated on the ability of a team to win a game of wheelchair basketball.

**TABLE 4:** Final model illustrating the estimated regression coefficients, standard errors, *p*-values and ORs for the intercept variable and for each action variable in a CPV.

	**Estimate**	**SE**	***z* value**	***p***	**OR**	**OR (95% CI)**	
						**Lower**	**Upper**
(Intercept)	0.309	0.272	1.137	0.255	1.363	0.800	2.328
xGame Status (a)							
Losing	–0.894	0.164	–5.448	$0.000^{*,**}$	0.409	0.296	0.563
Winning	0.858	0.166	5.178	$0.000^{*,**}$	2.359	1.701	3.259
Stage (b)							
Ranking Game	–0.568	0.153	–3.723	$0.000^{*,**}$	0.567	0.420	0.764
Quarter-Final	–0.430	0.115	–3.736	$0.000^{*,**}$	0.651	0.519	0.815
Semi-Final	–0.069	0.162	–0.423	0.672	0.934	0.680	1.285
Medal Match	–0.631	0.148	–4.266	$0.000^{*,**}$	0.532	0.398	0.711
Offensive Unit – 1.0–1.5 (c)							
Zero Players	1.658	0.370	4.479	$0.000^{*,**}$	5.247	2.601	11.170
Two Players	0.557	0.265	2.101	0.036^*^	1.745	1.045	2.956
Offensive Unit – 2.0–2.5 (d)							
Zero Players	–1.265	0.323	–3.916	$0.000^{*,**}$	0.282	0.149	0.527
Two Players	0.143	0.195	0.735	0.462	1.154	0.789	1.694
Offensive Unit – 3.0–3.5 (e)							
Zero Players	–2.219	0.477	–4.648	$0.000^{*,**}$	0.109	0.042	0.273
One Players	–1.639	0.349	–4.694	$0.000^{*,**}$	0.194	0.095	0.376
Three or More Players	1.801	0.368	4.899	$0.000^{*,**}$	6.056	2.973	12.568
Offensive Unit – 4.0–4.5 (f)							
Two Players	1.860	0.394	4.723	$0.000^{*,**}$	6.424	3.029	14.257
Three Players	–1.111	0.399	–2.785	0.005^∗∗^	0.329	0.150	0.719
Defensive Unit – 1.0–1.5 (g)							
Zero Players	–0.619	0.432	–1.433	0.152	0.538	0.223	1.224
Two Players	3.067	0.756	4.056	$0.000^{*,**}$	21.486	5.170	101.914
Defensive Unit – 2.0–2.5 (h)							
Zero Players	–2.115	0.741	–2.852	0.004^∗∗^	0.121	0.026	0.488
Two or More Players	2.227	0.743	2.995	0.003^∗∗^	9.268	2.291	42.959
Defensive Unit – 3.0–3.5 (i)							
Zero Players	–2.582	1.182	–2.185	0.029^*^	0.076	0.007	0.709
One Player	–1.461	0.499	–2.927	0.003^∗∗^	0.232	0.085	0.604
Three or More Players	3.046	0.767	3.971	$0.000^{*,**}$	21.035	4.931	101.503
Defensive Unit – 4.0–4.5 (j)							
Two Players	0.934	0.475	1.968	0.049^*^	2.546	1.023	6.623
Three Players	1.708	0.863	1.978	0.048^*^	5.519	1.058	31.685
Start of Possession (k)							
Free Throw	0.543	0.229	2.372	0.018^*^	1.721	1.097	2.692
Inbound – Baseline	–0.008	0.098	–0.077	0.938	0.992	0.819	1.202
Inbound – Endline	0.405	0.203	1.999	0.046^*^	1.500	1.010	2.238
Offensive Rebound	0.348	0.156	2.232	0.026^*^	1.417	1.045	1.926
Other Start	–0.003	0.511	–0.007	0.995	0.997	0.369	2.807
Sideline – Back	0.527	0.178	2.970	0.003^∗∗^	1.694	1.200	2.408
Sideline – Front	0.329	0.154	2.130	0.033^*^	1.389	1.028	1.883
Turnover	0.320	0.176	1.818	0.069^+^	1.377	0.977	1.948
End of Possession (l)							
Basket Scored When Fouled	–0.111	0.280	–0.396	0.692	0.895	0.518	1.557
Defensive Rebound	–0.169	0.096	–1.758	0.079^+^	0.845	0.700	1.020
Foul Against	–0.034	0.234	–0.146	0.884	0.966	0.612	1.534
Foul For	0.268	0.128	2.087	0.037^*^	1.307	1.017	1.683
Free Throw	–0.532	0.258	–2.064	0.039^*^	0.587	0.356	0.979
Handling Error	–0.562	0.174	–3.236	0.001^∗∗^	0.570	0.405	0.801
Offensive Rebound	0.157	0.145	1.086	0.277	1.170	0.882	1.556
Other	0.603	0.246	2.452	0.014^*^	1.827	1.136	2.982
Out of Bounds	0.045	0.150	0.297	0.766	1.046	0.781	1.404
Violation Against	-0.045	0.269	-0.169	0.866	0.956	0.565	1.623
Defensive System (m)							
1 Man Press	0.177	0.203	0.872	0.383	1.194	0.802	1.780
2 Man Press	0.346	0.144	2.409	0.016^*^	1.413	1.067	1.876
3 Man Press	0.527	0.154	3.427	0.000^***^	1.694	1.255	2.294
4 Man Press	0.371	0.210	1.766	0.077^+^	1.450	0.962	2.193
5 Man Press	0.083	0.228	0.366	0.714	1.087	0.696	1.701
Highline	0.519	0.129	3.016	0.000^***^	1.681	1.306	2.169
No Defensive System	0.204	0.201	1.015	0.310	1.227	0.828	1.826

^+^*p* < 0.1, ^*^p < 0.05, ^∗∗^*p* < 0.01, ^∗∗∗^*p* < 0.001; B, estimate coefficient; SE, standard error; OR, odds ratios; CI, confidence intervals. The baseline categories within the intercept were (a) Drawing; (b) Pool Stage; (c) One Player; (d) One Player; (e) Two Players; (f) One Player; (g) One Player; (h) One Player; (i) Two Players; (j) One Player; (k) Defensive Rebound; (l) Basket Scored; (m) Zone.

For the CPV Game Status, taking Drawing as the reference category, when a team changed to a status of Winning (as would be expected) the odds of finally winning the game were increased (OR: 2.359; 95% CI: 1.701–3.259; *p* < 0.001), whilst (again as would be expected) a fall in the odds of winning was registered when the status changed to Losing (OR: 0.409; 95% CI: 0.296–0.563; *p* < 0.001). The Stage CPV, highlighted that the reference category of Pool Stage resulted in the highest odds ratio with the various different knock-out rounds of the tournament registering lower odds ratios (Ranking Game: OR: 0.567; CI: 0.420–0.764; *p* < 0.001; Quarter-Final: OR: 0.651; CI: 0.519–0.815; *p* < 0.001; Semi-Final: OR: 0.934; CI: 0.680–1.285; *p* = 0.672; Medal Match: OR: 0.532; CI: 0.398–0.711; *p* < 0.001).

The number of 1.0–1.5, 2.0–2.5, 3.0–3.5 and 4.0–4.5 players involved in the offensive and defensive teams presented differences in the odds ratios. The reference categories for both the offensive team and the defensive team were One 1.0–1.5 player, One 2.0–2.5 player, Two 3.0–3.5 players and One 4.0–4.5 player. From an offensive perspective, it was observed that reducing the number of 1.0–1.5 players from One to Zero registered a higher odds ratio (OR: 5.247; 95% CI: 2.601–11.170; *p* < 0.001), increasing the number of 2.0–2.5 players from One to Two registered a slightly higher odds ratio (OR: 1.154; 95% CI: 0.789–1.694; *p* < 0.001), increasing the number of 3.0–3.5 players from Two to Three or More registered higher odds ratio (OR: 6.056; 95% CI: 2.973–12.568; *p* < 0.001) and increasing the number of 4.0–4.5 players from Zero or One to Two registered higher odds ratios ratio (OR: 6.424; 95% CI: 3.029–14.257; *p* < 0.001).

Whilst observing the defensive team, it was found that increasing the number of 1.0–1.5 players from One to Two resulted in a higher odds ratio (OR: 21.486; 95% CI: 5.170–101.914; *p* = 0.152), increasing the number of 2.0–2.5 players from One to Two or More registered a higher odds ratio (OR: 9.268; 95% CI: 2.291–42.595; *p* = 0.003), increasing the number of 3.0–3.5 players from Two to Three or More registered a higher odds ratio (OR: 21.035; 95% CI: 4.931–101.503; *p* < 0.001) and increasing the number of 4.0–4.5 players from Two to Three resulted in a higher odds ratio (OR: 5.519; 95% CI: 1.058–31.685; *p* < 0.05). However, due to the total classification points a team can have on-court teams would be restricted by some of these findings. Thus, the highest likelihood of winning a game of wheelchair basketball was achieved when the defensive team comprised of Two 1.0–1.5 players and Three 4.0–4.5 players (OR: 118.510), whereas an offensive team that comprised of Two 2.0–2.5 players and Three 3.0–3.5 players had the highest odds ratio (OR: 36.670).

When considering the Start of Possession CPV, Free Throw exhibited the highest odds ratio (OR: 1.721; 95% CI: 1.097–2.692; *p* < 0.05). Possessions that began within the defensive team’s half of the court (Inbound – Endline: OR: 1.500; 95% CI: 1.010–2.238; *p* < 0.05; Offensive Rebound: OR: 1.417; 95% CI: 1.045–1.926; *p* < 0.05) or from an inbound just inside the offensive team’s half (OR: 1.389, 95% CI: 1.028–1.883; *p* < 0.05) demonstrated odds ratios greater than one. Possessions that ended from a Handling Error had a negative impact on the ability of a team to win a game (OR: 0.570, 95% CI: 10.405–0.801; *p* < 0.001). Whilst, when the referees were required to stop the game, for example, due to a player being unable to push themselves back up after being tipped over, a higher odds ratio was registered (OR: 1.827, 95% CI: 1.136–2.982; *p* < 0.05).

Table 4 also showed that the effect of the opposition changing the Defensive System from Zone to either a 2 Man Press, 3 Man Press or a Highline was found to significantly increase the odds of the offensive team winning the game (2 Man Press: OR: 1.413, 95% CI: 1.067–1.876; *p* < 0.05; 3 Man Press: OR: 1.694, 95% CI: 1.255–2.294; *p* < 0.001; Highline: OR: 1.681, 95% CI: 1.306–2.169; *p* < 0.001).

McFadden, Cox and Snell, and Nagelkerke Pseudo-*R*^2^ values were calculated to compare the maximum likelihood of the developed model against a null model (Table 5). The pseudo *R*^2^ values presented indicated that the model does explain at least a reasonable amount of the variation in game outcomes. These values do have an upper limit of 1 when the model would explain all the variation but these Pseudo-*R*^2^ values are well known for very rarely attaining values near this upper limit, even for well-fitting models (Heinzl and Mittlböck, 2003). The regression equation, derived from the model, was used for predicting the accuracy of the binary logistic regression model against the 30% out of sample testing data (1,844 possessions). An area under the ROC curve value of 0.759 was established for the model when predicting the possessions within the sample testing data. The model was therefore considered acceptable (Hosmer and Lemeshow, 2000) at accurately predicting win probabilities in elite men’s wheelchair basketball.

**TABLE 5:** Pseudo-*R*^2^ values comparing the binary logistic regression model to a null model.

**Pseudo-*R*^2^ measure**	**Pseudo-*R*^2^ value**
McFadden *R*^2^	0.158
Cox and Snell *R*^2^	0.192
Nagelkerke *R*^2^	0.260

## Discussion

The aim of this paper was twofold, firstly, to identify the key variables associated with team success in elite men’s wheelchair basketball and secondly to explore the impact of each key action variable upon the outcome of performance through the use of binary logistic regression modeling. Fifteen CPVs were found to relate to Game Outcome, with Game Status being identified as the most important CPV due to achieving the largest chi-square value and lowest *p*-value. The final model demonstrated the sequential action variables within the classification units CPVs and Game Status CPV had the largest impact on predicting Game Outcome. The Stage of the competition was also found to be an important factor, but this simply seemed to be explained by the fact that during the later stages, the teams that eventually win dominate possession less during the games as the quality of opposition increases. The findings from this study largely support previous research regarding stage of competition (van Rooyen et al., 2008, 2010; O’Donoghue et al., 2016), defensive structure (Gómez et al., 2006; Tenga et al., 2010) and in-game status (Lago-Peñas and Dellal, 2010; Marcelino et al., 2011; Almeida et al., 2014) in relation to the importance of game outcome.

Findings from previous wheelchair basketball studies have highlighted the importance of 4.0 and 4.5 classification players in relation to achieving higher CBGS scores and assisting toward a positive game outcome (Vanlandewijck et al., 2004; Gómez et al., 2015a), however, the findings in this study regarding classification challenge these. The findings around player classification identified the chance of winning is reduced if “Three” 4.0 or 4.5 players were used during a possession by odds of 0.329. Playing with three 4.0 players restricts the classification of each of the remaining two players on-court to 1.0 in order to stay within the 14-point total team classification score. Players who are classified as 1.0 players have been found to regularly achieve low or negative CBGS scores (Gómez et al., 2015a), typically accumulating higher frequency counts regarding the number of times possession is lost, the number of fouls committed and turnovers in comparison to other classification groups (Vanlandewijck et al., 1995). These players have limited trunk function in the forward plane and no active rotation which significantly impairs balance in both forward and sideways directions, impairing their pushing, dribbling, passing and shooting performance (Perriman, 2014). The players typically fulfil the role of a screen as they are reliant on the wheelchair for support in all planes of movement and are susceptible to losing the ball (Vanlandewijck et al., 1995). Thus, the model developed found that having two 1.0 classified players on the court and three 4.0 players at the same time was found to negatively impact the odds of success, due to the impairment of handling, pushing and shooting of the low classified players. This finding was identified through the produced odds ratio in the model (OR: 0.575) in comparison to the reference line-up of one 1.0–1.5 player, one 2.0–2.5 player, two 3.0–3.5 players and one 4.0–4.5 player. As highlighted, 1.0 players typically perform other roles that are not included within the CBGS, for example blocks and picks, however, the authors of this paper argue that if higher classified players perform these roles, in addition to their current role, they offer the team a higher offensive threat and would therefore increase the odds of success.

In contradiction to the work of Molik et al. (2009) who found similarities between low class players (classification 1.0–3.0) and low game efficiency, the model demonstrated that playing with “Three or More” Offensive Unit – 3.0–3.5 players on the court improves Game Outcome (OR: 6.056, 95% CIs: 2.973–12.568, *p* < 0.001). Subsequently, the limitations of physical movement associated with 1.0 players can be overcome by a combination of players with the following classification: 2.0, 2.5, 3.0 or 3.5 (OR: 36.670). These findings align with Vanlandewijck et al. (2004) who discovered that players who have a classification between 2.0 and 3.5 have similar technical abilities. However, within International Wheelchair Basketball Federation’s classification system, Perriman (2014, p. 9) briefly stated that class 2.0 players have “partially controlled trunk movement in the forward plane, active upper trunk rotation but no low trunk function [and] no controlled sideways movement” whereas class 3.0 players have “good trunk movement in the forward plane, good trunk rotation [and] no controlled trunk movements sideways.” Although, the good or partially controlled movements in the forward plane and the ability to engage all or some of the trunk for rotation enable both classification groups to hold the ball outstretched without inclining the head or trunk, return to an upright position with minimal effort when pushing, rotate the upper trunk to receive a pass from behind and are able to lean forward when shooting to propel the ball toward the basket (Perriman, 2014), and thus have a similar functional capacity. As a result, the ability to have some form of partial control in the forward and vertical planes could be argued to make them superior players to those with a 1.0 classification (Gil-Agudo et al., 2010). However, both these classification groups have limited sideways plane movement and are unable to incline to one side, unlike 4.0 or 4.5 players. Although, Gómez et al. (2014, 2015a) identified that similar performances are observed for players between adjacent classes, in particular around the mid-class players.

Furthermore, playing with a greater number of Offensive 3.0–3.5 players than Offensive 4.0–4.5 players could be due to 3.0 player’s being able to sit 5 cm higher in the chair in comparison to 3.5, 4.0 and 4.5 players. These additional 5 cm would provide players, if they elect to position the top of the cushion at a height of 63 cm in comparison to 58 cm from the floor (International Wheelchair Basketball Federation [IWBF], 2014b), with a potential advantage when being defended in the act of shooting or rebounding. Santos et al. (2014) highlighted the importance of trunk stability and movement when completing faster movement directions in the forward plane when rebounding. van der Slikke et al. (2016) also noted that international players average seat height is significantly higher to enable players to achieve greater upward reach. van der Woude et al. (1989) found that a higher cushion height negatively impacted a players turning capacity due to shift in the start and end position of the hand on the push rim. However, considerable changes in chair design have occurred since this research was published and thus the findings may be different. Coaches and players, therefore, are required to consider a player’s seating height in relation to the role on the court and their capacity to execute the fundamental basketball movements of shooting, rebound the ball and turning. However, it is important to note that no anthropometric data was collected by the authors and that could influence a player and teams capabilities to execute the fundamental wheelchair basketball skills.

In addition, the capacity of mid-point classification players (class 2.0, 2.5, 3.0 and 3.5 players) to propel the chair has been found to be superior in both straight-line speed and weaving than low-class players (class 1.0 and 1.5) (Crespo-Ruiz et al., 2011). Therefore, our current findings support the notion that coaches need to carefully consider when and how they use class 1.0 and 1.5 players within line-ups. The odds ratios produced for different line-ups can be used to highlight the benefits of a team comprising of more 3.0–3.5 players than 4.0–4.5 players and using these players in the on-court five in relation to game outcome. These findings provide useful information for coaches to carefully consider the line-up configurations of players, acknowledging the strengths and limitations of certain classifications in order to identify an optimum line-up. Of course, this information also adds further debate around the classification system in wheelchair basketball and whether it is fit for purpose. Consideration, therefore, needs to be taken regarding the individual’s technical characteristics and subsequently could challenge some of the findings and interpretations around classification and line-up configurations. However, it is important to note that technical and tactical characteristics of players do not depend on classification, as classification is “based on the player’s physical capacity to execute fundamental basketball movements.” The findings highlighted within this study also align with the International Wheelchair Basketball Federation [IWBF] (2019b) recent decision to introduce a more stringent review process. The revised process involves additional stages for new player’s to ensure the sport’s classification philosophy continues to be in agreement with the International Paralympic Committee Classification Code and that of the Paralympic Games.

The model also illustrated the chances of winning a game during the knock-out stages of the 2015 European Wheelchair Basketball Championships are more testing than winning a “Pool” stage game. O’Donoghue et al. (2016) found as teams advanced through a competition the points difference between the two teams decreased along with the probability of winning. Gómez et al. (2015a) also observed this trend in wheelchair basketball because the quality of opposition increased during each stage of the tournament. The researchers found that players in teams that finished in the top four teams achieved higher shooting efficiencies and CBGS scores than other players in lower ranked teams. This finding supports the notion that you have to win knock-out games, and achieve higher scores, and you do not have to win pool games to stay in the competition. However, as the analyzed event was a qualification tournament for the Paralympics, the increased points difference observed during the semi-final stage provided evidence to suggest teams were content with qualification as winning the quarter-finals automatically qualified the teams for the Rio de Janeiro Paralympic Games.

Furthermore, the 10-day wheelchair basketball tournament may have affected the probability of winning due to players becoming fatigued and thus leading to a reduction in skill execution. Montgomery et al. (2008) found this occurred during a three-day basketball tournament, reporting small to moderate impairments in players’ performance due to physical fatigue. Lertwanich (2009) also found 1.0 players were susceptible to becoming physically fatigued at a quicker rate than amputees due to the impartment of sweating and vasomotor control. These findings, therefore, reiterate the importance of line-up combinations and minimizing the use of 1.0 players in an attempt to maintain consistent performances, especially in the later stages of a tournament. However, without recording the cardiovascular and locomotion demands of these players during the tournament it is unknown whether fatigue affected the odds ratios achieved. Thus, future international tournaments should incorporate cardiovascular and locomotion demands in addition to the sport performance analysis data to collate a broader picture of performance. However, the results of this study have clearly indicated that Stage, and thus the quality of opposition, affect Game Outcome.

Game Status was found to be a significant CPV in relation to Game Outcome (OR: 2.359) more than double if the team started a possession in a state of “Winning.” Therefore, suggesting that the game winner can be predicted in wheelchair basketball earlier in the game in comparison to running basketball. This may be as a result of the NBA being a much more closely contested competition with longer breaks in between games (Horowitz, 2018), allowing for mental and physical recovery, than wheelchair basketball tournaments. It is important to note that rule adaptions have been made between wheelchair basketball and basketball, specifically relating to dribbling the ball and controlling the chair, and thus differences exist. However, the fundamental principles of the sport are the same and due to the limited existing knowledge within wheelchair basketball, basketball literature was drawn on to inform the discussion of this research. The increasing odds of winning the game, both in basketball and in wheelchair basketball highlight the importance of shooting effectiveness, however, no shooting related CPVs were presented in the final model. Despite this, Shot Location was included in the model developed using the automated forward selection approach and thus indicates it is potentially an important CPV.

The defensive system operated by the opposition in this research was found to significantly affect a team’s ability to score more points, and thus win a game. The results found that the tighter and more structured the defensive system, the harder it was for the offensive team to break down the system and score points. Research surrounding dynamical systems theory confirms this interpretation (Reed and Hughes, 2006; Gréhaigne and Godbout, 2014; Araújo and Davids, 2016). Gómez et al. (2006) found within Spanish basketball Playoffs’ series, the losing team found it difficult to break down a Zone defensive system and convert possession into points. By operating a zonal defensive system the attacker-defender dyads are closer and thus the available space is less. Therefore, restricting the ability of the attackers to create an open shot, which has been found to increase the shooting efficiencies of players (Zhang et al., 2017). The findings from the developed model confirmed this finding, highlighting that the odds of winning were greater than one when a Highline system was adopted as well as a number of the pressing systems. Recent technology advancements in terms of radio-frequency-based indoor (Rhodes et al., 2015), bluetooth-based systems (Figueira et al., 2018) or artificial intelligence (Kristan et al., 2009) could allow for more objective data in relation to defensive systems and attacker-defender dyads to be collected that could inform future practice. On the other hand, this finding may be the result that zonal type defenses are the most trained and developed systems in team sports (Gómez et al., 2015b). Despite these ideas for future exploration, the results from our analyses indicate that the defensive system, space and pressure are important factors for coaches to consider in training and when devising game strategies to prevent opponents from scoring and capitalizing on disorganized defensive systems (Tenga et al., 2010).

In agreement with capitalizing on disorganized defensive systems, possessions that began from an offensive rebounds or from a turnover registered odds ratios greater than one. These findings align with those of Sampaio et al. (2007) who found that if a team was able to capitalize on offensive rebound The results demonstrated if a team are in a state of winning when the possession started the probability of winning the game increases. Similarly, Gómez et al. (2016) identified that the starting quarter score in the fourth quarter was significantly related to final points differential in the NBA. The researchers found that teams who were ahead during the first possession of the fourth quarter were almost twice as likely to win the game (OR: 1.75). Although this point may seem obvious in relation to Game Outcome, the odds of winning the game utilizing the wheelchair basketball data were of a team to establish a lead and maintain the lead has been explored in a number of team sports in relation to the concept of momentum (see Hughes et al., 2015). The findings presented here agree with the work of Gómez et al. (2016) whereby the importance of capitalizing on each ball possession and ensuring individual players are able to convert under pressure shooting opportunities into points affects the final outcome. their likelihood of winning increased, although this was restricted to play-off games. Similarly, Gómez et al. (2006, 2010) identified that when teams adopted a zonal man-to-man defensive system a higher number of turnovers were generated, subsequently increasing the likelihood of gaining field goal positions nearer to the basket following a successful transition from defense into offence. Additionally, significant differences and odds greater than one were found for Inbound – Endline, Sideline – Front, Sideline – Back and other starts. Schmidt et al. (1999) speculated that this was not uncommon, as offensive teams in basketball look to exploit set-plays from these locations. Teams typically run “backdoor plays” where the offensive player attempts to free up space by initially moving toward their own basket before quickly turning to exploit the vacant space. The aim of these pre-planned patterns of play is to target a specific point on the playing area or an individual to try and provide a platform to open up space for team mates to attack (Barkell et al., 2016). This tactic is observed within wheelchair basketball. Once a defensive player has followed the ball player, other offensive players would use their chair to restrict the progress of defensive players in returning to a rigid defensive shape and allow the offensive player with the ball a less pressurized shooting opportunity. Thus, these findings reinforce the need for teams to have specific strategies for possessions that start from inbounds but also from open play situations that enable the creation of optimal space-time opportunities that enable players to take shooting opportunities under less pressure (Lamas et al., 2011; Fonseca et al., 2012, 2013).

## Conclusion

This study has identified the key determinants of team success in elite men’s wheelchair basketball that can contribute to a game-winning performance. The model indicated that the capacity to have the optimal line-up on the court who can score and prevent an opponent from scoring by creating defensive pressure is a key component in predicting winning odds. In addition, consideration needs to be taken regarding how team’s play from possession that start from within the defensive team’s half, as reducing the space between the attacker-defender dyads and the basketball was associated with lower ORs. The most significant and important finding from the model is regarding game status and maintaining a winning margin on the scoreboard. The application of these findings emphasizes the importance of key tactical and technical abilities of the players regarding the on-court decision making processes in the act of shooting. However, it is important to note that this study has focused on the performances of the qualified teams from the 2015 European Wheelchair Basketball Championships and thus different nations playing styles during different international competitions may not be transferable. Furthermore, variables associated with how team’s advanced the ball toward the basket, including the number of passes and the number of players who handled the ball in the possession, were not recorded.

Despite this, this study is the first to have identified the key variables associated with team success and explored the impact of each key action variable upon the outcome of performance through the use of binary logistic regression modeling. These findings offer some practical implications to coaches, players and support staff to assist the players’ learning and decision-making skills and enhance their likelihood of winning based on the accumulation of optimal CPV sequences. The information obtained regarding classification line-ups can be used to effectively improve a team’s ability to win a game of wheelchair basketball and during the selection of players prior to major international tournaments. The information can also be used when planning training and game strategies to take advantage of an opponent’s tactical strategies and assist team’s with maximizing the ability to establish and maintain a lead throughout a game.

## Data Availability

The dataset generated for this study can be found in the Worcester Research and Publications collection (https://eprints.worc.ac.uk/id/eprint/7551).

## Ethics Statement

Following the granting of ethical approval from the University of Worcester’s Ethics and Research Governance Committee, written, voluntary informed consent, was obtained from the host nation to access the required game footage.

## Author Contributions

JF devised the structure of the manuscript, collected and analyzed the data, and drafted the manuscript. AO provided guidance and support for statistical analyses and reporting, and commented on the final draft. DP devised the structure of the manuscript, oversaw the whole research process, commented on drafts, and approved the final draft.

## Conflict of Interest Statement

The authors declare that the research was conducted in the absence of any commercial or financial relationships that could be construed as a potential conflict of interest.

## Supplementary Material

MATERIAL S1 Data set for team performances during the 2015 European Wheelchair Basketball Championship.
